# Poly(furfuryl alcohol) as a Surface Modifier for Cellulose Nanocrystals Reinforced HDPE Nanocomposites

**DOI:** 10.1021/acs.biomac.6c00329

**Published:** 2026-07-07

**Authors:** Marlon Bender Bueno Rodrigues, Mariane Weirich Bosenbecker, Amanda Dantas de Oliveira, Silvana I. Wolke, Willian Cézar Nadaleti, Neftali Lenin Villarreal Carreno, Rafael de Avila Delucis, André Luiz Missio

**Affiliations:** † Graduate Program in Materials Science and Engineering (PPGCEM), 37902 Federal University of Pelotas (UFPel), Pelotas 96010-610, Brazil; ‡ Chemistry Institute, 28124 Federal University of Rio Grande do Sul (UFRGS), Porto Alegre 91501-970, Brazil; § Graduate Program in Environmental Sciences (PPGCAmb), Federal University of Pelotas (UFPel), Pelotas 96010-610, Brazil

## Abstract

To overcome the thermodynamic incompatibility between hydrophilic cellulose nanocrystals (CNCs) and nonpolar polyolefins without relying on traditional fossil-based or solvent-heavy covalent compatibilizers, this study introduces a fully biobased, noncovalent strategy. We propose the *in situ* aqueous polymerization of poly­(furfuryl alcohol) (PFA) to create a hydrophobic core–shell morphology around CNCs, enabling efficient melt-blending with high-density polyethylene (HDPE). Morphological and spectroscopic analyses confirmed the successful physical encapsulation, preserving the nanocrystals’ structural integrity. Consequently, the PFA-modified nanocomposites exhibited robust interfacial adhesion, achieving a 42% increase in Young’s modulus (1856 MPa) alongside enhanced specific strength. Furthermore, the PFA shell acted as an effective thermal barrier, increasing the CNC degradation onset from 250 to 360 °C, and enhanced matrix crystallinity by 9.7%. This facile, green modification distinguishes itself by bridging the polarity gap without toxic solvents, offering a highly scalable route for sustainable structural biocomposites.

## Graphical abstract

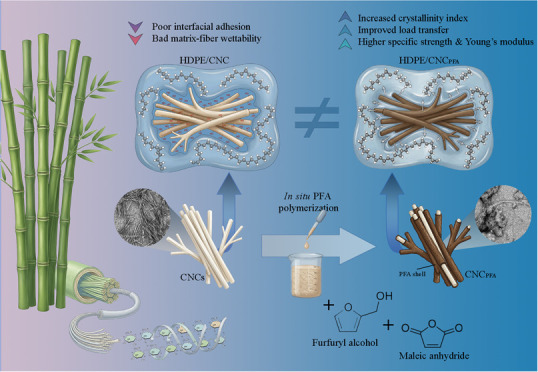

## 1 Introduction

Incorporation of high aspect ratio nanoparticles in polyolefin matrices is a viable strategy to fabricate strong and tough nanocomposites, with functional properties depending on the chosen nanomaterials. Carbon nanotubes, silver nanowhiskers, and plant-based nanomaterials such as cellulose nanofibrils (CNF) and crystals have been used. The latter are plant-based nanoparticles that can contribute to increasing the renewable content in materials, while also introducing gains in mechanical properties. Cellulose nanocrystals (CNCs), which are obtained from the acid hydrolysis of plant fibers—such as bamboo, owing to its rapid growth and high availability—are shorter and more rigid colloids when compared to their fibrillar counterparts (i.e., CNF), which facilitates their dispersion during melt processing. Regardless of the nanoparticle nature and intrinsic properties, interfacial adhesion is still a major challenge to be overcome within the (bio)­nanocomposite field. Poor interfaces between this reinforcing nanomaterial and polymeric matrices (e.g., high–density polyethylene (HDPE), polypropylene, epoxy) take place due to the non compatible adhesion interaction between hydrophilic reinforcements/fillers and the hydrophobic matrices.^1^ Therefore, effective nanocompositing strategies should account for favorable interfacial interactions between the two components, which is obtained mostly by (i) addition of coupling agents, and (ii) surface modification of the nanoparticle.^2,3^

However, while some interfacial compatibilizers improve performance, they are not derived from plant-based sources, which poses a sustainability concern. Additionally, their use increases the production cost of these composites, which does not solve the economic challenges driving the investigation, development, and application of biocomposites. The most common industrial approach for compatibilization involves the use of compatibilizers such as maleic anhydride-grafted polyethylene (MAPE) or block copolymers like PLMA-*b*-PHEMA during melt processing.^4^ While these agents can improve adhesion to a certain degree, they are typically fossil-based, posing a sustainability concern that contradicts the purpose of using bionanofillers. Alternatively, precise surface chemical modifications allow for the design of the interface at a molecular level. “Grafting-from” techniques, which involve the immobilization of initiators on the cellulose surface to grow polymer chains, have shown great promise. For instance, ring-opening polymerization using tin octoate as a catalyst has been used to graft hydrophobic polymers, such as poly­(ε-caprolactone), directly onto CNC hydroxyl groups, creating a “hairy” layer that is miscible with polyethylene.^5^ Similarly, atom transfer radical polymerization (ATRP) has offered alternative routes for compatibilization. ATRP has, for example, been reported for grafting styrene and methyl methacrylate hydrophobic chains onto CNCs to tailor their interfacial compatibility.^6^ Besides, grafted copolymers require the addition of a third polymeric phase that does not inherently protect cellulose from thermal degradation during processing, whereas traditional covalent modifications often rely on toxic organic solvents, complex multistep reactions, and harsh conditions that can compromise the intrinsic crystallinity of the CNCs, making them difficult to scale and economically unfeasible for commodity plastic applications. Thus, there is a clear need for a facile, solvent-free, and biobased modification strategy that can effectively compatibilize the CNC-HDPE interface.

In this context, we propose the surface modification of CNCs with poly­(furfuryl alcohol) (PFA) as a novel, robust, and sustainable compatibilization strategy, studied for modifying lignocellulosic products due to its pseudopositive and negative regions that can possibly induce synergistic interactions between filler and matrix.^7,8^ Furfuryl alcohol (FA) is a renewable monomer derived from the reduction of furfural, which is widely produced from agricultural byproducts rich in pentosans, such as sugar cane bagasse or corncobs.^9^ FA is distinguished by its unique reactivity; under acidic conditions and elevated temperatures, it undergoes polycondensation to form a dark, cross-linked, and hydrophobic biopolymer resin.^10^ This behavior presents a synergistic opportunity for CNC modification: the residual sulfate ester groups on acid-hydrolyzed CNC surfaces create a naturally acidic environment that can catalyze or facilitate the in situ polymerization of FA. Although the impregnation of wood with FA (furfurylation) is a mature technology for improving dimensional stability and durability, the use of FA to encapsulate nanoscale cellulosic fillers for polymer reinforcement is surprisingly underexplored. Although the application of PFA as a surface modifier in thermoplastic nanocomposites is an emerging field, its effectiveness in modifying lignocellulosic reinforcements has been validated in other material classes. For instance, in fiber-cement composites, the furfurylation of natural fibers has led to toughness increments of approximately 100% and notable improvements in flexural strength due to reinforced interfacial bonding.^11^ Furthermore, PFA modification of wood flour in polyurethane foams has demonstrated a reduction in water uptake by as much as 200%, highlighting the potential of this treatment to mitigate the hydrophilic nature of biomass-derived fillers.^12^ We hypothesize that by polymerizing FA directly onto the CNC suspension prior to drying, a rigid PFA shell will form around the nanocrystals. This coating is expected to serve a dual function: First, it sterically stabilizes the nanocrystals, preventing the hydrogen bonding that leads to hornification during drying; second, the hydrophobic nature of the PFA shell enhances thermodynamic compatibility with the nonpolar HDPE matrix. In this context of HDPE-based nanocomposites, the formation of a PFA shell on CNCs is expected to serve as a hydrophobic barrier and promote a mechanically stable interface, targeting improvements in tensile modulus and thermal stability superior to those achieved with unmodified CNCs. These measurable targets serve as the primary metrics for evaluating the effectiveness of the proposed in situ polymerization strategy. Rather than optimizing a specific commercial formulation, the primary objective of this foundational proof-of-concept study is to investigate whether the *in situ* aqueous polymerization of PFA can serve as an effective, green, and physical compatibilizing strategy to resolve the severe thermodynamic incompatibility between hydrophilic CNCs and nonpolar HDPE matrices at representative low filler loadings.

## 2 Materials and Methods

### 2.1 Materials

Sodium hypochlorite (NaClO), sodium hydroxide (NaOH), and sulfuric acid (H_2_SO_4_) were obtained from Dinâmica Química Contemporânea (Brazil). FA and maleic anhydride (MA) were obtained from Sigma-Aldrich. All reagents were used without additional purification. HDPE—grade GM9450F, density of 0.952 g/cm^3^, melt flow index (at 190 °C/5 kg) of 0.33 g/10 min—was supplied by Braskem (Brazil).

### 2.2 Obtaining Cellulose Nanocrystals

CNCs were extracted from bamboo biomass (*Bambusa vulgaris*). The biomass was dried to a constant mass, milled in a knife mill, and particle size was adjusted using a 32-mesh sieve. This milled biomass was treated with a 10% NaOH solution (w/v) at 80 °C for 1 h, filtered, and washed with distilled water until reaching neutral pH, bleached with a 20% NaClO solution (w/v) for 60 min, filtered again with distilled water, and dried in an oven for 24 h at 50 °C. This bleaching procedure followed established protocols for *B. vulgaris* biomass to ensure the removal of residual lignin and hemicelluloses prior to acid hydrolysis.^13,14^ Subsequently, CNCs were produced through acid hydrolysis performed at 45 °C for 1 h in a 35% H_2_SO_4_ solution. The biomass was mixed into the solution at a mass/volume ratio of 1:20. Cold distilled water was then added to the solution, followed by centrifugation at 3600 rpm for 10 min. The centrifuged solution was transferred to dialysis membranes (Spectra/POR6MWCO = 3500 g·mol^–1^) immersed in water until a neutral pH was reached. The obtained suspensions were prefrozen in an ultralow temperature freezer Indrel Scientific IULT335D (Indrel Scientific, Brazil) at −80 °C for 24 h and subsequently lyophilized to obtain the dry powdered nanomaterials. The freeze-drying process was conducted in a Liotop L202 (Liobras, Brazil) at a temperature of −40 °C under a vacuum pressure of 300 μm Hg for 48 h to ensure the complete sublimation of ice and the acquisition of a fine, dry powdered nanomaterial.

### 2.3 PFA Polymerization and Nanocomposite Processing

The obtained CNC powder was added to distilled water, and a 3% (w/v) suspension was prepared in an UltraTurrax disperser at 5000 rpm for 8 min. The modification of the obtained CNCs was carried out by adding FA to the suspension at a concentration of 50% (relative to the mass of CNC in the suspension). Additionally, 5% MA relative to the mass of FA was incorporated into the suspension, followed by manual mixing for 5 min and heating at 80 °C for 24 h to initiate polymerization and the formation of PFA on the surface of the CNCs.^15^ To separate the modified nanocrystals from unreacted furfuryl alcohol monomers, maleic anhydride, and unbound water-soluble PFA oligomers, the suspension was subjected to a rigorous purification protocol. The mixture was thoroughly washed with distilled water through successive centrifugation cycles until the supernatant became clear and neutral, ensuring the complete removal of free residues prior to composite incorporation. The purified modified nanocrystals were then frozen and subsequently freeze-dried. MA was employed as an acid source to catalyze the in situ polymerization of FA. Upon introduction to the aqueous CNC suspension, MA hydrolyzes into maleic acid, initiating the formation of the PFA shell on the nanocrystal surfaces. This catalyst was chosen for its established compatibility with polyolefin systems, although it serves a strictly catalytic role in the current process.

The processing of nanocomposites was carried out through melt blending using a single-screw extruder (AXPlásticos, Brazil) with an L/D ratio of 20 operating at a constant screw speed of 70 rpm, which corresponded to an estimated material residence time of approximately 60 to 90 s within the extruder barrel. To ensure consistent polymer melting while minimizing premature thermal exposure, the temperature profile across the extruder zones from the feed hopper to the die was strictly maintained at 170 °C, 180 °C, and 190 °C. HDPE provided by Braskem S/A was used as the polymer matrix. The samples produced were called HDPE/CNC1%, HDPE/CNC_PFA_1%, HDPE/CNC_PFA_1.5%, and neat HDPE. The quantities of reinforcement in the sample names, given in percentages, refer to the amount of filler relative to the total mass in the produced materials.

While MAPE is the established industry benchmark for polyolefin composites, this research prioritizes the fundamental investigation of PFA as a sustainable, biobased modifier. A direct performance comparison was considered beyond the scope of this initial study, which focuses on the *in situ* polymerization mechanism and interfacial properties of the PFA-modified CNCs. However, the findings provide a solid scientific basis for future benchmarking against traditional synthetic agents under standardized processing conditions.

### 2.4 Characterization

#### 2.4.1 CNCs’ Characterization

The thermal degradation profile of the produced samples was investigated using thermogravimetric analysis (TGA), including the derivative thermogravimetric curve (DTG). The test was conducted from 30 to 600 °C at a heating rate of 15 °C·min^–1^ with an argon flow of 50 mL·min^–1^. The equipment used was the TGA-1000 (Navas).

Functional groups present in the cellulose, CNCs, and CNCs polymerized with FA were investigated using Fourier-transform infrared (FTIR) spectroscopy. The samples were predried and analyzed in a Shimadzu IRPrestige-21 spectrophotometer over a range of 500–4000 cm^–1^, with a total of 45 scans and a resolution of 2 cm^–1^, utilizing attenuated total reflectance (ATR) mode.

The solid–state cross–polarization magic angle spinning carbon-13 nuclear magnetic resonance (CP-MAS 13C-NMR) spectra were recorded on an Agilent 500 MHz (model DD2), using MAS rates of 10 kHZ at frequency of 125.69 MHz. The samples were packed in 4 mm diameter zirconia rotors. Chemical shifts are reported relative to the signals of adamantane used as external standard. The spectra were obtained with 20,000 scans, 2.55 μs pulse width, 0.8 ms of contact time, 35 ms acquisition time, and 2.5 s of recycle delay.

To assess the morphology of the nanomaterials, the powdered samples were redispersed in distilled water and ultrasonicated for 30 min. Subsequently, a droplet of the resulting suspension was deposited onto carbon-coated copper grids and allowed to stand for 10 min. The samples were then negatively stained with a 2% uranyl acetate solution for another 10 min and dried in a desiccator to ensure solvent evaporation. The images were acquired using a JEOL JEM-1400 transmission electron microscope (TEM) operating at an acceleration voltage of 100 kV.

The morphological features and dimensions of the isolated neat CNC and CNC_PFA_ particles were evaluated using a field emission gun scanning electron microscope (FEG-SEM, TESCAN MIRA4 LMS). Prior to analysis, the nanoparticle samples were dried on conductive stubs and subjected to gold sputtering for 30 s using a Quick Coater Sanyu Electron SC-701. The FEG-SEM micrographs were acquired under high vacuum with an accelerating voltage of 30 kV.

#### 2.4.2 Nanocomposites’ Characterization

Thermogravimetric and FTIR analyses were repeated for nanocomposites using the same parameters as previously described.

Measurements of tensile strength, elastic modulus, elongation at break, and toughness were obtained following the ASTM D638 standard for type IV specimens, with five specimens for each composition. Tests were performed using a universal testing machine (Emic DL200) equipped with a 1 kN load cell, at a tensile test speed of 5 mm·min^–1^, and samples were deformed until failure. The apparent density of the composites was assessed using a digital caliper and a Shimadzu ATX224 analytical balance with four decimal places. All samples were dried to constant mass before measurement and weighing.

Subsequently, to investigate the internal structure and filler dispersion within the consolidated materials, the polymer composites were cryo-fractured by immersion in liquid nitrogen (approximately −200 °C). The fracture surfaces of the composites were then gold-coated and examined using a Shimadzu SSX 550 scanning electron microscope (SEM) operating at an acceleration voltage of 15 kV.

The wettability of the composite surfaces was investigated using the sessile drop method, performed with a TL100 Theta Lite tensiometer and OneAttension software. Distilled water droplets (10 μL) were deposited on the nanocomposites, and the contact angle between the droplets and the surface was monitored for 60 s, with five repetitions per sample. The samples were previously dried in an oven at 60 °C to constant mass.

The samples were subjected to differential scanning calorimetry (DSC) analysis in a Q20 calorimeter (TA Instruments) under nitrogen flow to determine the thermal characteristics of the composites (such as melting temperature (*T*_m_), crystallization temperature (*T*_c_), and degree of crystallinity (*X*_c_)). Initially, the samples were heated from 30 to 200 °C at a heating rate of 10 °C per minute, remaining at this temperature for 4 min to erase the thermal history related to material processing. Subsequently, cooling was performed at the same rate down to 30 °C (to obtain information on material crystallization), followed by a final heating to 200 °C (to obtain melting information). The crystallinity degree of the nanocomposites was determined using eq 1.^16^

1 $$X_{c}=\frac{\Delta H_{m}}{{\Delta H}_{f}^{0}\cdot \left(1-\omega \right)}\times 100\%$$

Where *X*_c_ is the crystallinity index, Δ*H*_*m*_ (J·g^–1^) is the melting enthalpy of the sample, Δ*H*_*f*_^0^ is the theoretical melting enthalpy of 100% crystalline HDPE (286.7 J·g^–1^), and ω is the weight percentage of the reinforcements in the composite composition.^17,18^

Thermal analysis (TGA and DSC) and spectroscopy data were conducted on a single representative sample for each composition studied. To ensure the representativeness of the results and mitigate potential intersample variability, a homogenization protocol was implemented for thermal and spectroscopic characterizations. For TGA, DSC, and FTIR analyses, multiple specimens from each composite group were ground into a fine powder and thoroughly mixed to form a single representative composite sample. This pooling approach was specifically designed to minimize the impact of localized structural differences and ensure that the measured parameters, including crystallinity, enthalpy, and thermal degradation temperatures, represent the average properties of the homogenized material batches. All quantitative experimental results are presented as the mean value ± standard deviation (SD). For mechanical characterizations, the mean and SD were calculated from a minimum of five independent test specimens per composite formulation. In all graphical representations, the error bars explicitly denote the standard deviation of the mean. Data were subjected to variance analysis (ANOVA). When the null hypothesis was rejected, Fisher’s multiple comparison test—known as the Least Significant Difference (LSD) test—was used to compare means and detect significant differences. All statistical tests were conducted at a significance level of 0.05 (*p* < 0.05). These procedures were performed using the Statgraphics Centurion 19 software.

## 3 Results and Discussion

### 3.1 CNCs’ Analysis

Figure 1A presents the infrared spectra of treated and untreated CNC. To ensure accurate qualitative comparison and eliminate intensity artifacts caused by varying sample-to-crystal contact pressure, all ATR-FTIR spectra were normalized and processed using a Savitzky-Golay smoothing filter with a second polynomial order and a 50-point window. At 1030 cm^–1^, bands related to the C–O–C linkages at the C3 position of cellulose were detected^19^ and/or C–O–C glycosidic linkages,^20^ with an increase in the band for CNC (compared to cellulose), suggesting minimal loss of cellulosic material mass during the acid hydrolysis process. Consequently, the decrease in this band for CNC_PFA_ indicates a reduction in such linkages in the cellulose molecule. At 1160 cm^–1^, the wavelength responsible for exciting vibrations attributed to C–OH in the C-6 plane is also depressed for CNC_PFA_.^21^

**Figure 1:**
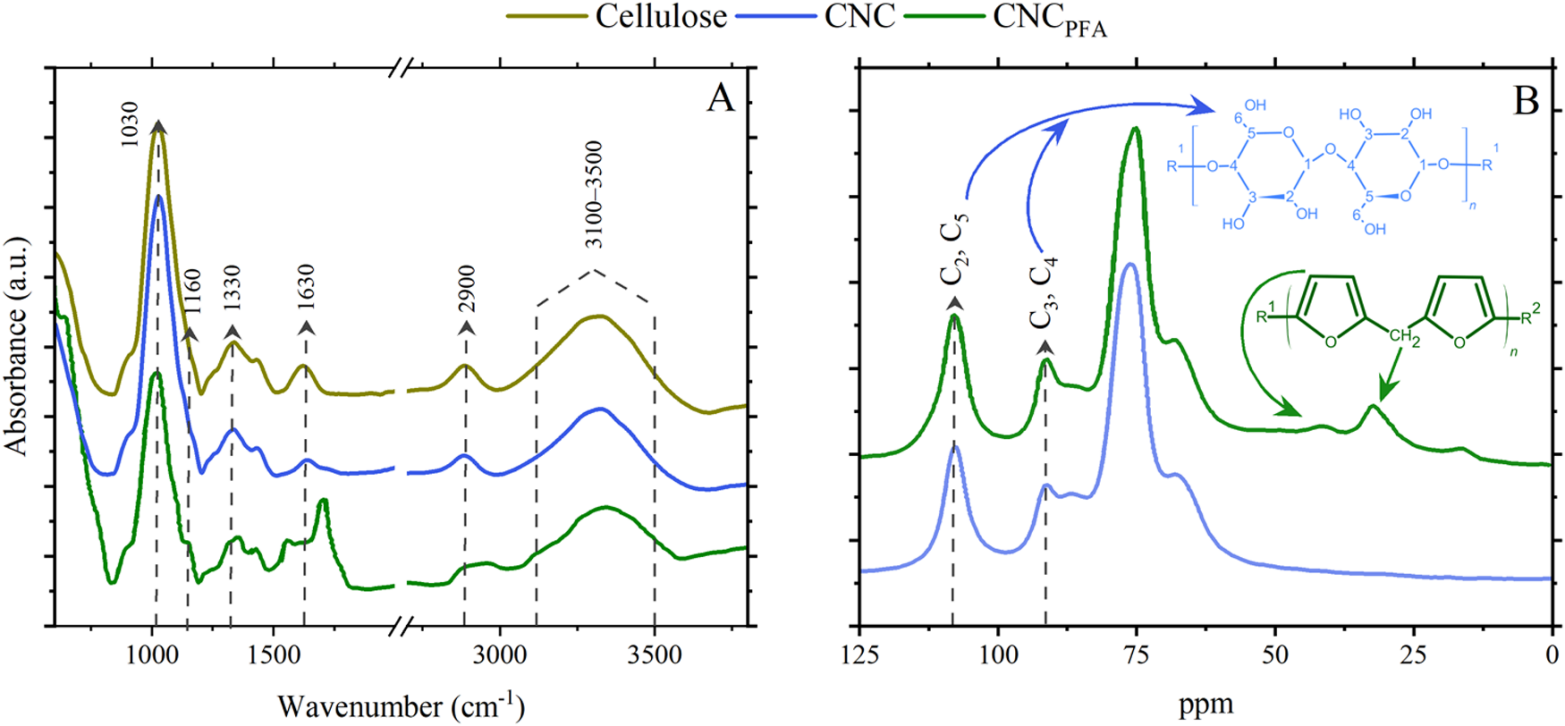
(A) FTIR spectra, and (B) 13C–CPMAS spectra of cellulosic materials.

In the 1330 cm^–1^ region, the planar bending vibration associated with OH was detected,^22^ although this region is often linked to the bending of CH groups and the balancing of CH in rings.^23^ The CNC_PFA_ spectrum in this region also showed decreases, with only a short band at 1700 cm^–1^.

For cellulose and CNCs, the peak at 1630 cm^–1^ corresponds to the bending of CH_2_–O–H linkages, as well as H–O–H vibrations from water absorbed by the material.^24^ A distinct behavior is the presence of a nonprominent peak near 1700 cm^–1^ for the CNC_PFA_ spectrum, which has been discussed in the literature as being caused by the stretching of CO in carboxylic acid groups as well as aromatic CO molecules.^25^ Free carbonyls in carboxylic acids and/or in aromatic rings are not found in the chemical structure of cellulose or in PFA. Therefore, two hypotheses are raised to explain the origin of the peak at 1700 cm^–1^: (i) A chemical reaction between CNCs and FA generates cross-linking and dissolves other functional groups, and (ii) the generation of compounds through hydrolytic cleavage of the furan ring of FA facilitated by the aqueous medium in which the polycondensation reaction was cultivated.^26^ Concurrently, the emergence of the nonprominent peak near 1700 is attributed to carbonyl (C=O) stretching vibrations.^27^ While MA hydrolyzes to maleic acid in an aqueous environment and features carboxylic carbonyl groups that absorb in this exact region, the modified nanocrystals were rigorously washed multiple times exclusively with distilled water via successive centrifugation cycles to guarantee the complete removal of free, unreacted catalyst residues. Consequently, this band primarily arises from carbonyl functionalities generated through the hydrolytic cleavage and ring-opening reactions of the FA monomers during the *in situ* polymerization process in the aqueous medium. This indicates that the peak reflects an intrinsic structural feature of the complex, water–polymerized PFA shell encapsulating the cellulose nanocrystals rather than the physical presence of loose chemical impurities.

Near 2900 cm^–1^, bands corresponding to the vibrational stretching of – CH, – CH_2_, and – CH_3_ groups,^14^ present in lignocellulosic fibers are found in the form of symmetrical and asymmetrical methyl and methylene groups in side chains.^28^ The decrease of this band for CNC_PFA_ may also relate to the utilization of such side groups in cross-linking reactions with PFA or to the complete impregnation of CNCs by PFA (masking these molecules from the spectroscopic analysis)—which would confirm the affinity between the materials. Finally, in the region between 3000–3500 cm^–1^, the bands referring to OH vibrations characteristic of cellulosic molecules are identified.^28,29^

^13^CP-MAS NMR spectroscopy was employed as a fundamental technique to elucidate the nature of the interface between CNC and PFA (Figure 1B). The spectrum of unmodified CNCs exhibits the well-established characteristic signals for cellulose of the type I allomorph.^30^ The signal at approximately 108 ppm is attributed to the C1 carbon, which participates in the β-1,4-glycosidic linkage between anhydroglucose units. The glycosidic linkage carbon (C4) region is particularly informative about the supramolecular structure of cellulose. Two distinct resonances are observed: a sharp signal at 91 ppm, associated with carbons located in the crystalline interior of the nanocrystals, and a broader signal at 87 ppm, attributed to carbons in amorphous domains or on the crystal surface.^30^ A broad, overlapping signal, centered at approximately 75 ppm, encompasses the carbons of the pyranose ring that contain secondary hydroxyl groups, the C2, C3, C5 cluster.^31^ At last, a prominent signal at 68 ppm represents the exocyclic C6 carbon, which carries the primary hydroxyl group (−CH_2_–OH). This signal is of fundamental importance for the present analysis, as the C6-OH group is the most sterically accessible and chemically reactive site on the cellulose surface, making it the most likely location for any grafting reaction, such as the formation of an ether bond.^32^

The structure of PFA is notoriously complex and dependent on polymerization conditions. The polymerization of FA in an acidic medium, especially under the aqueous and heated conditions used in this work, is accompanied by a series of side reactions that significantly modify the final polymer structure. The predominant reaction is the polycondensation of FA units through methylene bridges (−CH_2_−), which preferentially connect the C5 position of one furan ring to the methylene group at the C2 position of another ring.^33^ In the CNC_PFA_ spectrum, the substituted C2 and C5 carbons resonate at 153 ppm, while the unsubstituted C3 and C4 carbons appear at 108 ppm. The main signal for methylene bridges (−CH_2_−) is observed at 32 ppm, while methine groups (−CH−) resulting from branching appear at 41 ppm.^34^

The aqueous and acidic reaction conditions promote reactions that alter the PFA structure, generating new chemical functionalities. The presence of CNCs, with their residual acidic surface from H_2_SO_4_ hydrolysis, can locally catalyze these transformations. The presence of water in an acidic medium facilitates the hydrolytic cleavage of the furan ring, a well-documented side reaction that leads to the formation of carbonyl species, such as γ-diketones.^34^ The definitive evidence for this reaction is the presence of a low-intensity signal at 208 ppm in the CNC_PFA_ spectrum, which is the characteristic assignment for the carbonyl carbon in diketone structures. Ring opening can also lead to the formation of γ-lactone structures, typically at the ends of the polymer chain.^28^ The CNC_PFA_ spectrum confirms the presence of these species through signals at 175 ppm (lactone carbonyl carbon) and 16 ppm (lactone methyl group carbon).

The hypothesis of covalent grafting between PFA and CNC would imply the formation of a chemical bond, most likely an ether linkage involving the primary hydroxyl group at C6, which is the most reactive. Such a reaction would induce a predictable change in the chemical environment of the C6 carbon, which would be detectable by NMR. The formation of an ether bond (Cellulose–C6–O–CH_2_–PFA) would result in an electronic deshielding of the C6 carbon. The etherification of a primary alcohol causes a downfield shift of the α-carbon signal (in this case, C6) by several ppm.^35^ Therefore, one would expect to observe a significant decrease in the intensity of the original C6 signal at 68 ppm, accompanied by the appearance of a new signal, corresponding to the etherified C6 (C6′), at a higher chemical shift, typically in the 70–75 ppm region. The absence of a clear C6′ signal and the maintenance of the original C6 signal constitute the most robust spectroscopic evidence that large-scale etherification did not occur. This conclusion is strongly corroborated by studies in the literature that have investigated analogous reactions. Notably, Shen et al.^36^ used model compounds—cellobiose for cellulose and dihydroconiferyl alcohol for lignin—to study the reactions with PFA. Their 13C NMR analysis conclusively demonstrated that no reaction occurred between PFA and cellobiose, while clear condensation reactions were observed with the lignin model. This work establishes an important precedent, indicating that PFA reacts preferentially with lignin over cellulose, which is consistent with the observations of the present study. Although an extremely low degree of grafting, below the detection limit of the technique, cannot be completely ruled out, such a minor mechanism would not be sufficient to explain the improvements observed in the thermal and mechanical properties of the composites, to be discussed.

Beyond the macroscopic improvements in thermomechanical performance (later discussed), the proposed PFA core–shell modification aligns robustly with the fundamental principles of green chemistry and the circular economy. The *in situ* polymerization of PFA is conducted entirely in an aqueous medium under relatively mild conditions, inherently eliminating the emission of volatile organic compounds and drastically reducing the process’s environmental footprint. Furthermore, FA is a completely renewable monomer derived from the catalytic conversion of hemicellulose found in abundant agricultural waste streams. By utilizing a water-based reaction system and sustainable lignocellulosic precursors, this modification strategy bypasses the reliance on fossil-based compatibilizers and presents a highly scalable, economically viable pathway for the large-scale industrial manufacturing of advanced thermoplastic biocomposites.

Figure 2 shows the thermal degradation profiles of the cellulosic raw materials obtained in this study. Three traditional stages of mass loss were identified in the thermogravimetric analysis.

**Figure 2:**
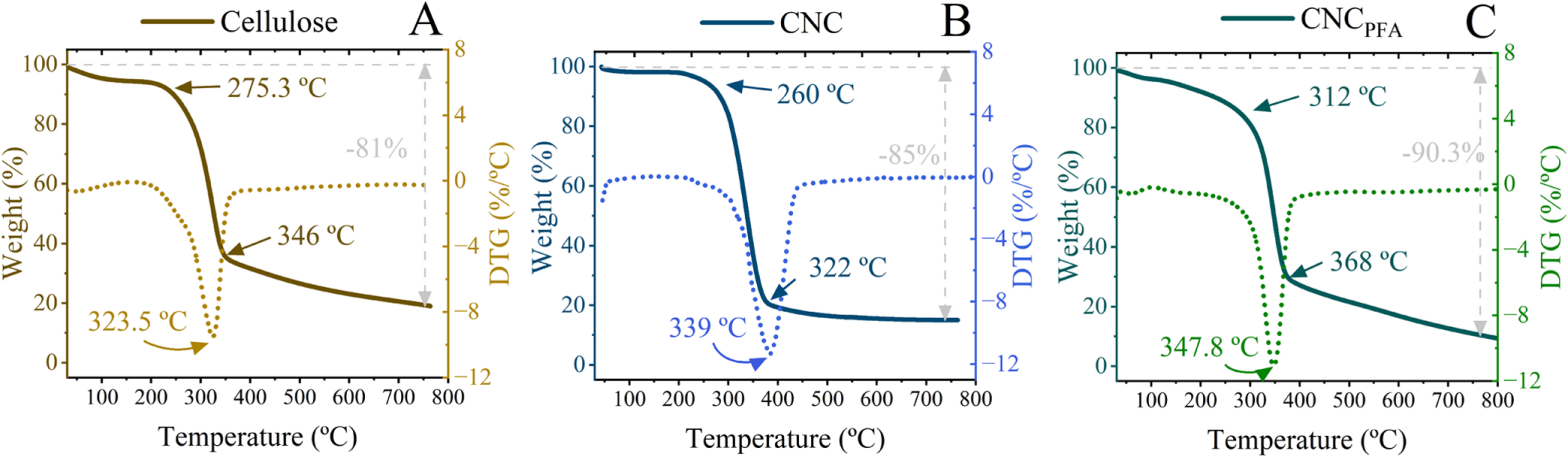
TGA and DTG curves of (A) cellulose, (B) CNC, and (C) CNC_PFA_. Onset, endset, DTG maxima temperatures, and total mass loss are highlighted.

The first mass loss event (I) occurred approximately between 20 and 220 °C and is associated with the evaporation of moisture and volatile residues generated during the processes of obtaining cellulose, CNC, and CNC_PFA_. Notably, the steep incline of the thermogravimetric curve for CNC_PFA_ in this region may be attributed to two possible factors: (i) The greater total concentration of polar groups in CNC_PFA_, responsible for increasing the moisture content of the sample and/or (ii) oligomeric residues and low molecular weight molecules resulting from the polymerization process of PFA.^37^

The second stage of mass loss (II) occurred between 240 and 360 °C, being the event with the highest percentage loss for all samples: 57.2%, 60.31%, and 75.66% for cellulose, CNC_PFA_, and CNC, respectively. This episode is primarily characterized by the degradation of α-cellulose, the depolymerization of cellulosic chains marked by decarboxylation and the deterioration of glycosidic units.^38^ The higher thermal sensitivity of CNCs has been a topic previously addressed in the literature, related to a significantly lower activation energy for thermal degradation due to the acids used in the acid hydrolysis process.^39^ The large surface area of CNCs compared to common cellulose may also contribute to explain this occurrence.^14^

Still in the second thermal event, it is observed that the impregnation of PFA in CNCs contributed to the decrease in mass loss in this temperature range (240–360 °C), providing greater thermal stability to the material. The thermal decomposition reactions of PFA are described by the cleavage of methylene linkages and the formation of compounds such as 2-methyl furan and 2-furfuryl-5-methylfuran.^15^ Furthermore, furan linkages are also degraded, resulting in volatile compounds such as acetone, 2-butanone, and 2-pentanone,^40^ elucidating the progressive mass loss of CNC_PFA_ in the third stage as a function of the temperature increase.

Finally, the third stage of mass loss (III), starting from 380 °C, indicates the near-complete pyrolysis of cellulose, leaving behind higher molecular weight carbonized molecules in the form of ash. Here, CNCs maintain a virtually constant mass percentage (17%)—a value in agreement with previous records in the scientific literature,^41^—while CNC_PFA_ shows only lower residual mass levels than CNCs starting from 635 °C, a temperature level that is rarely part of the conditions destined for cellulose nanostructures applications.

Through Figure 2, it is also possible to identify the temperatures at which thermal degradation peaks will occur for each of the cellulosic samples: 323.46 °C for cellulose, 337 °C for CNC, and 346.74 °C for CNC_PFA_. Supporting the results, the increase in the peak thermal degradation temperature of CNC_PFA_ suggests a minimally greater thermal stability of the nanocrystals modified with PFA. The enhancement in thermal stability, characterized by the shift in the onset of thermal degradation from approximately 260 °C for neat CNCs to 312 °C for the CNC_PFA_ nanostructures, is governed by a dual stabilization mechanism rather than covalent interfacial cross-linking. The highly cross-linked PFA shell provides exceptional initial thermal shielding by physically encapsulating the cellulosic core, thereby restricting direct heat transfer and hindering the release of volatile pyrolytic species into the environment.^42^ As the degradation progresses at higher temperatures, the inherently high char yield of the PFA network promotes the dynamic formation of a stable, dense carbonaceous protective layer around the nanocrystals.^43,44^ This persistent char barrier effectively insulates the underlying cellulose from further thermal exposure and suppresses the early stage catalytic dehydration pathways typically induced by residual surface sulfate groups, validating the robust protective nature of the physical core–shell architecture for extreme thermal processing environments.

TEM micrographs provide visual evidence of the structural modification undergone by the cellulose nanocrystals (Figure 3). While unmodified CNCs (Figure 3A–C) exhibit well-defined, needle-like structures with sharp edges and characteristic high aspect ratios, the morphology of CNC_PFA_ (Figure 3C,D) is markedly different. The PFA-treated samples display a diffuse, amorphous layer encapsulating the crystalline whiskers, appearing as darker contrast regions surrounding the cellulosic core. Although the distinct rod-like whisker morphology appears partially obscured in the CNC_PFA_ TEM micrographs, this visual effect is strictly attributed to the comprehensive physical encapsulation by the amorphous PFA shell rather than any degradation of the cellulosic core. The structural integrity and preservation of the cellulose crystalline domains following the acidic and thermal modification conditions are conclusively corroborated by the solid-state ^13^C CP-MAS NMR analysis. The retention of the characteristic chemical shifts assigned to the highly ordered regions of cellulose confirms that the intrinsic crystallinity of the nanostructures remains completely intact beneath the protective polymer coating.

**Figure 3:**
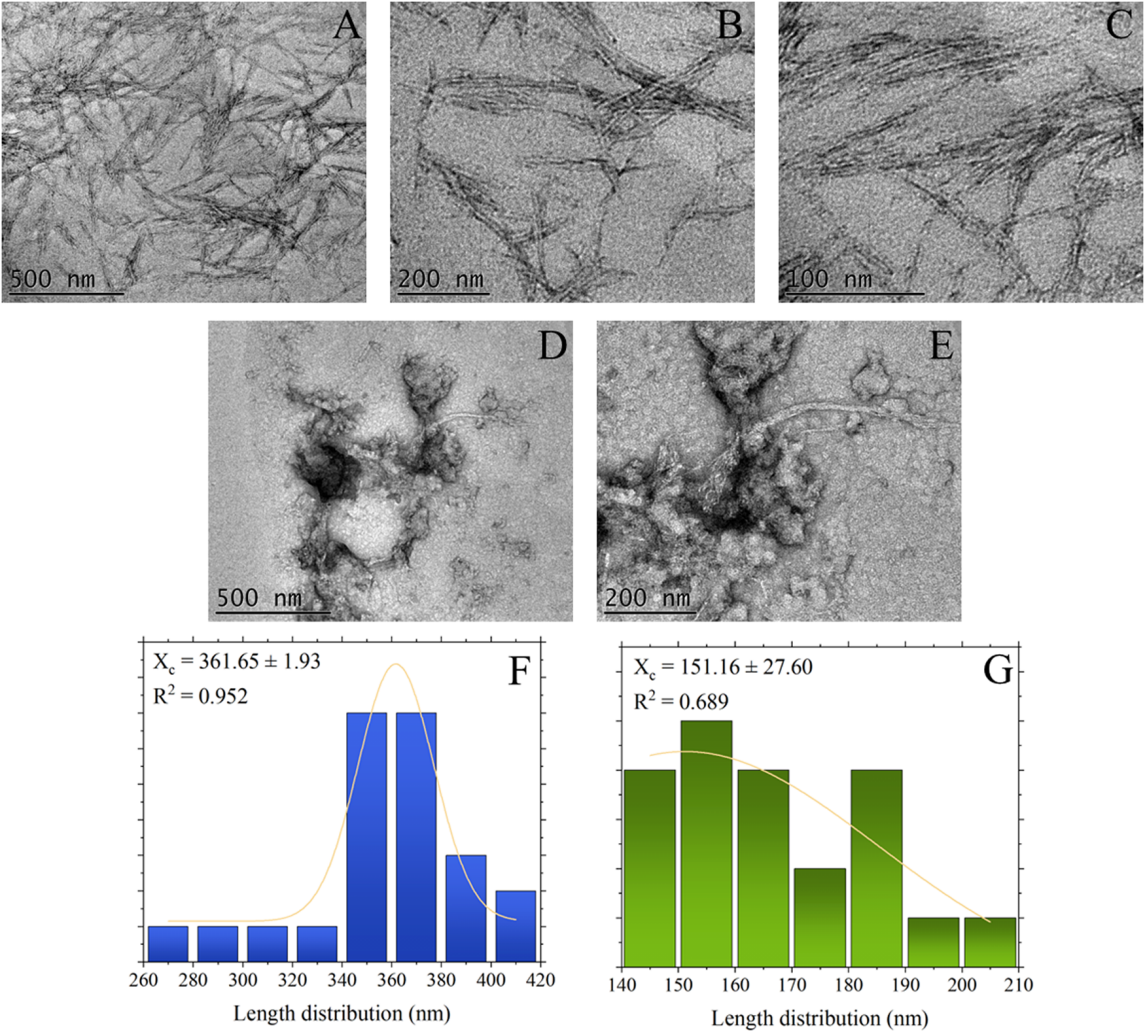
TEM images for (A–C) CNC, and (D, E) CNC_PFA_ at various magnifications. Corresponding size distribution histograms and fitted curves are shown for (F) the CNCs and (G) the CNC_PFA_ particles.

Furthermore, the TEM images confirm that the modification mechanism is primarily driven by *in situ* polymerization. The FA monomers polymerized around the CNC template, forming a dense, interpenetrating physical network or “shell” rather than a chemically grafted monolayer. This hydrophobic PFA shell is responsible for the enhanced compatibility with the HDPE matrix and the improved thermal stability observed in TGA, acting as a barrier that mitigates the hydrophilic nature of the nanofiller. It is important to note that the high visual prominence of the amorphous PFA phase in the TEM micrographs does not directly translate into dominant peak heights in the bulk spectroscopic analyses. This behavior is attributed to the disordered, cross-linked nature of the PFA shell, which inherently broadens both the infrared absorption bands and the solid-state ^13^C NMR resonances due to the wide distribution of local macromolecular environments and conformations. In contrast, the highly ordered, crystalline core of the cellulose nanocrystals produces extremely sharp, cooperative spectroscopic responses that visually dominate the spectra. Additionally, based on the initial formulation ratio (50 wt % of FA monomer relative to dry CNCs), the PFA network comprises a minority mass fraction of the total CNC_PFA_ nanostructure, which justifies why the structural features of cellulose remain spectroscopically prevalent despite the extensive physical encapsulation observed morphologically.

To quantitatively evaluate the dimensional features of the nanomaterials, particle size distributions (Figure 3F,G) were extracted from FEG-SEM images (Figure S1). The resulting histograms reveal an average length (*X*_c_) of 361.65 ± 1.93 nm for the neat CNCs. The high coefficient of determination (*R* ^2^ = 0.952) for the neat CNCs indicates a narrow, highly uniform distribution that closely follows a Gaussian profile. In contrast, the CNC_PFA_ exhibited a reduced average length of 151.16 ± 27.60 nm. Notably, the statistical fit for the CNC_PFA_ particles yielded a lower *R* ^2^ value (0.689), which quantitatively reflects a broader, more irregular, and heterogeneous length distribution. This dimensional variance and wider size distribution are likely consequences of the *in situ* polymerization process, which can lead to varying degrees of PFA coating thickness on individual crystals or induce partial localized interactions. Because the modification relies on the formation of a physical interpenetrating network rather than a controlled covalent monolayer, the degree of surface coverage is inherently heterogeneous. While not every individual CNC particle is uniformly encapsulated to the exact same degree, the bulk morphological (TEM), spectroscopic (NMR), and thermal (TGA) characterizations collectively confirm that the global modification efficiency is sufficiently robust to successfully shield the hydrophilic cores and bridge the polarity gap. Nevertheless, the process successfully maintained the nanometer-scale geometry of the fillers.

### 3.2 Nanocomposite’s Properties

Figure 4 shows the differences in thermal behavior among all produced samples, confirming the relevant role of CNC incorporation in the composites, as well as the modification performed on the fillers. Both items in the graph indicate the peak temperatures: For 4A, the crystallization temperatures (*T*_c_) are displayed, while 4B shows the melting temperatures (*T*_m_).

**Figure 4:**
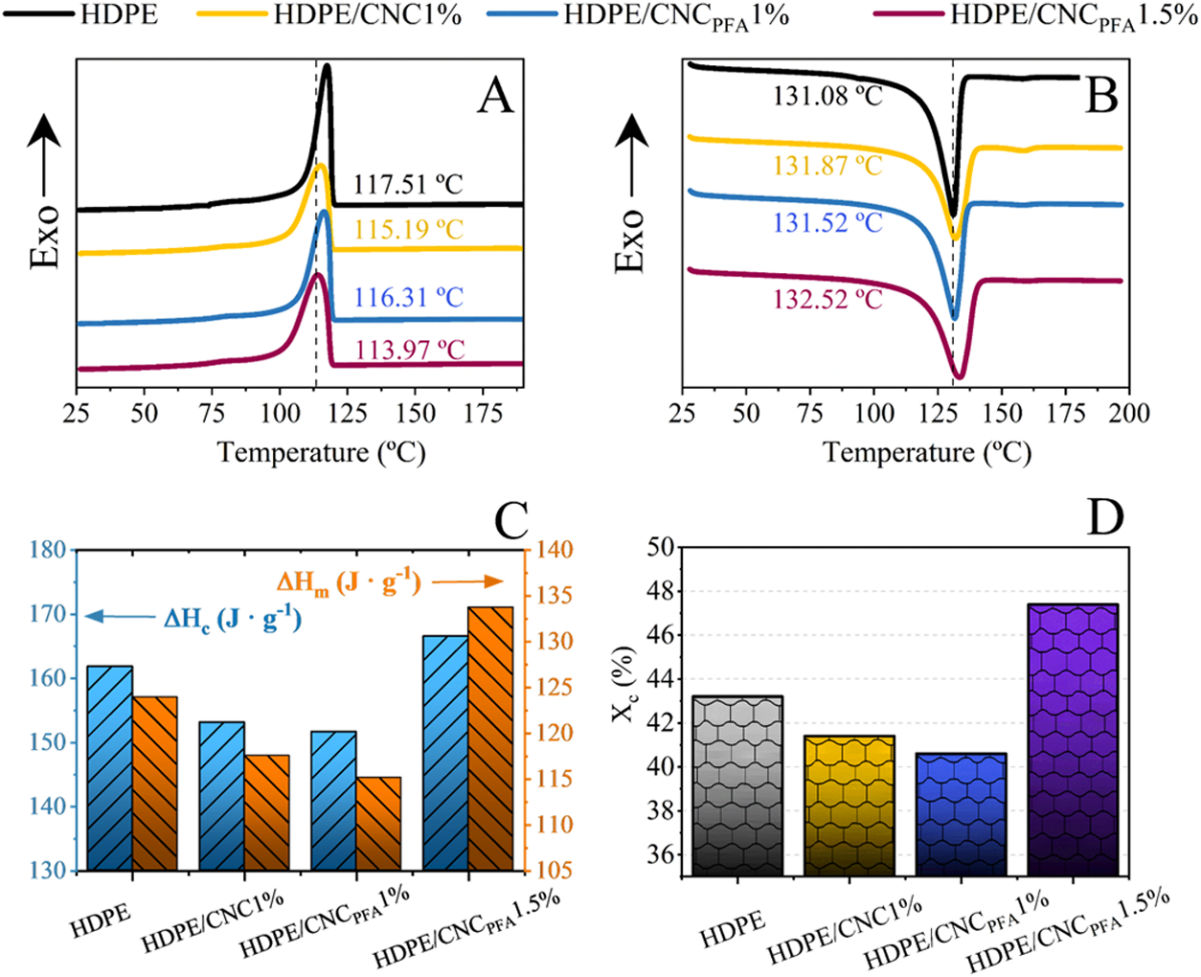
DSC curves for cooling (A) and heating (B), enthalpy parameters (C) and crystallinity index (D) of the nanocomposites.

The decrease in *T*_c_ of the nanocomposites was not accompanied by significant changes in *T*_m_, which barely depended on the modification of CNCs at a concentration of 1%. The graph shows that while the melting temperature of the samples did not present significant variations, the crystallization temperature did, especially for HDPE/CNC_PFA_1.5% (the lowest *T*_c_ recorded at 113.97 °C). This information supports the idea that at the concentration used, the chains grafted on the reinforcing nanoparticles reduced the mobility of HDPE chains, acting as physical barriers. This lower mobility would make it more difficult for the material to reorganize into a crystalline state, causing crystallization to occur at lower temperatures.

According to some authors, the *T*_c_ of polymer composites can decrease with the insertion of fillers due to improved compatibility between the polymer matrix and filler particles.^45^ This phenomenon is observed in poly­(ε-caprolactone) composites with acetylated cellulose nanocrystals and in poly­(ethylene oxide) composites with Na+ montmorillonite.^45,46^ The decrease in Tc is attributed to the inhibition of polymer crystallization near filler surfaces, which can promote noncrystalline conformations. However, the overall crystallization rate may increase due to additional nucleation sites in the bulk matrix.^46^ Although traditional nucleating agents often raise the *T*_c_, the observed decrease in this system suggests that the PFA-modified nanocrystals significantly restrict the segmental mobility of the HDPE chains at the interface, thereby delaying the reorganization of polymer chains into a crystalline lattice and requiring greater undercooling.

Figure 4C illustrates the values for melting enthalpy (Δ*H_m_*) and crystallization (Δ*H*_C_) of the nanocomposites, showing significant changes in the enthalpies of the produced samples. HDPE exhibited Δ*H*_C_ equal to 161.8 J·g^–1^ and Δ*H*_m_ of 123.9 J·g^–1^, values consistent with the literature.^18,47^ For HDPE/CNC1% and HDPE/CNC_PFA_1%, both Δ*H*_C_ (−54% and – 63%, respectively) and Δ*H_m_* (−51% and −7%, respectively) decreased. Again, it is noteworthy that HDPE/CNC_PFA_1.5% behaved differently from the other samples, with an increase (compared to HDPE) of Δ*H*_C_ by 29% and ΔH_m_ by 79%.

Correlating the enthalpy values and the crystallinity index of the nanocomposites, it is possible to verify that the melting and crystallization enthalpies are directly related to the crystalline phase of the obtained materials. It is observed that greater energy is required for both physical state transitions, which relates to the larger amounts of energy needed for the melting or crystallization of the crystalline fractions in the structure of the materials.^48^

The crystallinity of the nanocomposites shown in Figure 4D varied widely without displaying an overall trend. The addition of CNCs to the HDPE matrix caused reductions in *X*_c_, decreasing by −4.2% and −6% for HDPE/CNC1% and HDPE/CNC_PFA_1%, respectively, compared to HDPE. Nonetheless, an unexpected phenomenon was the increase in *X*_c_ for the HDPE/CNC_PFA_1.5% composite by 9.7% compared to HDPE. However, at sufficiently low concentrations, the restricted mobility of the HDPE polymer chains due to the presence of such reinforcements causes a decrease in the overall crystallinity of the composite, with the particles acting as barriers that prevent the formation of HDPE crystals.^49^ Furthermore, regarding HDPE/CNC_PFA_1.5%, despite the delayed onset of *T*_c_, the increased *X*_c_ confirms that PFA-modified CNCs act as effective nucleating agents by providing a high density of nucleation sites that ultimately leads to a higher overall crystalline fraction.^50,51^ While a detailed evaluation of the crystallization kinetics using Avrami-type modeling was not performed in the present study due to the requirement for multirate nonisothermal DSC analyses, the role of the CNC_PFA_ structures as highly efficient heterogeneous nucleating agents is robustly supported by the standard thermal data. These thermodynamic variations indicate that the physically encapsulated, hydrophobic PFA shell provides a highly compatible interfacial surface that successfully lowers the free energy barrier for polymer chain folding.

The decrease in crystallinity of polymer composites reinforced or filled with cellulose has been recorded in the literature. Kang & Kim (2023) incorporated microcrystalline cellulose (MCC) fibers into blends of HDPE and polylactic acid, varying the contents of the three constituents and verifying the resulting *X*_c_. For the analyzed cases, the incorporation of 3% (by weight) of MCC caused reductions in *X*_c_ from 17% to 13%. In another study, Khouaja et al. (2021) investigated the thermal and dielectric properties of HDPE composites with MCC and bleached Kraft pulp fibers. Following the trend of the previously cited work, reductions in *X*_c_ (from 70.9% for HDPE) were detected to 61.2%, 58.5%, and 57.1% in HDPE/MCC30%, HDPE/MCC40%, and HDPE/MCC50% composites, respectively.

Figure 5A shows the apparent contact angle of water droplets deposited on the surface of the nanocomposites over 60 s. Wettability studies involve the degree of wettability resulting from the interaction between a solid and a liquid, such as water. Generally, considering only the aspects related to secondary chemical interactions between liquids and solids, small contact angles (less than 90°) indicate high wettability surfaces, while larger contact angles correspond to low wettability.^52^

**Figure 5:**
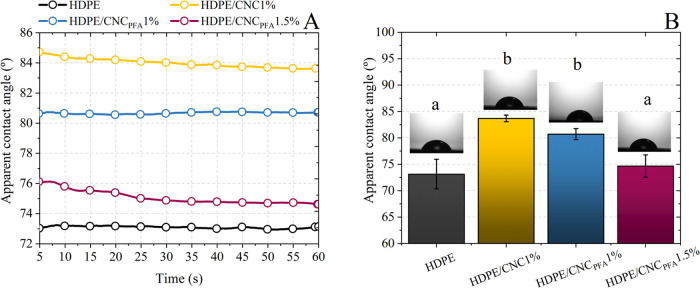
Kinetics (A) and value after 60 s (B) for the apparent contact angle of the composites. Error bars represent the SD calculated from independent replicates.

A notable behavior was the more hydrophobic surface of the composites reinforced with CNC compared to HDPE, a phenomenon unexpected due to the high density of polar functional groups present in CNCs,^53^ which have a strong affinity for water. An explanation for this occurrence is surface roughness (*R*_a_), an important factor in determining the hydrophobicity of materials. Rough surfaces can trap air molecules, which have a contact angle considered to be 180°.^52^ The possible agglomeration of CNCs, common in nanoscale particles, may have contributed to the increased surface roughness of the nanocomposites, which in turn caused higher contact angles.^54^ Kumar et al. (2021) functionalized CNCs through TEMPO (tetramethylpiperidine-1-oxyl) oxidation for surface application on glass fibers (GF) to improve their interfacial performance with polymer composites. In this study, the authors observed increases in Ra from 50 ± 40 nm (for their control sample) to 195 ± 11,5 nm (for the GF treated with oxidized CNCs). Statistically, the F ratio was 143.164, while the *p*-value was 0.0132. Thus, there is a statistically significant difference between the four variable means at a significance level of 5%. Furthermore, the furfurylation treatment of CNCs did not cause differences in surface behavior, as seen by the belonging of the means of the HDPE/CNC1% and HDPE/CNC_PFA_1% composites to the same homogeneous group of means in Figure 5B.

Although the increase in contact angle is theoretically attributed to surface roughness induced by filler agglomeration, direct quantitative topographical measurements (such as *R*_a_) were not performed in the present study. However, the formation of microagglomerates, as corroborated by the morphological SEM analysis, inherently introduces localized topographical irregularities that are widely reported in the literature to alter the wetting dynamics of thermoplastic biocomposites. Consequently, future studies dedicated to quantitative surface topography analysis using atomic force microscopy or optical profilometry on these PFA-functionalized materials would be highly beneficial to comprehensively elucidate the exact relationship between filler dispersion, surface roughness, and macroscopic water repellency.

The representative tensile stress–strain curves of the nanocomposites (Figure 6A) illustrate the direct mechanical response of the materials under load. Neat HDPE exhibited a characteristic ductile behavior. The addition of 1 wt % of CNCs (HDPE/CNC1%) resulted in an abrupt reduction in the elongation at break and overall toughness of the material (Figure 6E). This drop in deformation capacity and loss of ductility is a phenomenon classically reported in polymers reinforced with unmodified cellulose, occurring due to the strong tendency of the nanoparticles to agglomerate, which consequently creates stress concentration zones that trigger premature fracture.^55^ However, the surface modification with the PFA coating in the HDPE/CNC_PFA_1% and HDPE/CNC_PFA_1.5% composites promoted a notable recovery of the elongation at break and an improvement in toughness compared to the sample with CNCs. The presence of the polymeric shell indicated a more efficient particle dispersion and a reduction in critical interfacial defects, allowing the HDPE matrix to withstand higher stress levels before failure.

**Figure 6:**
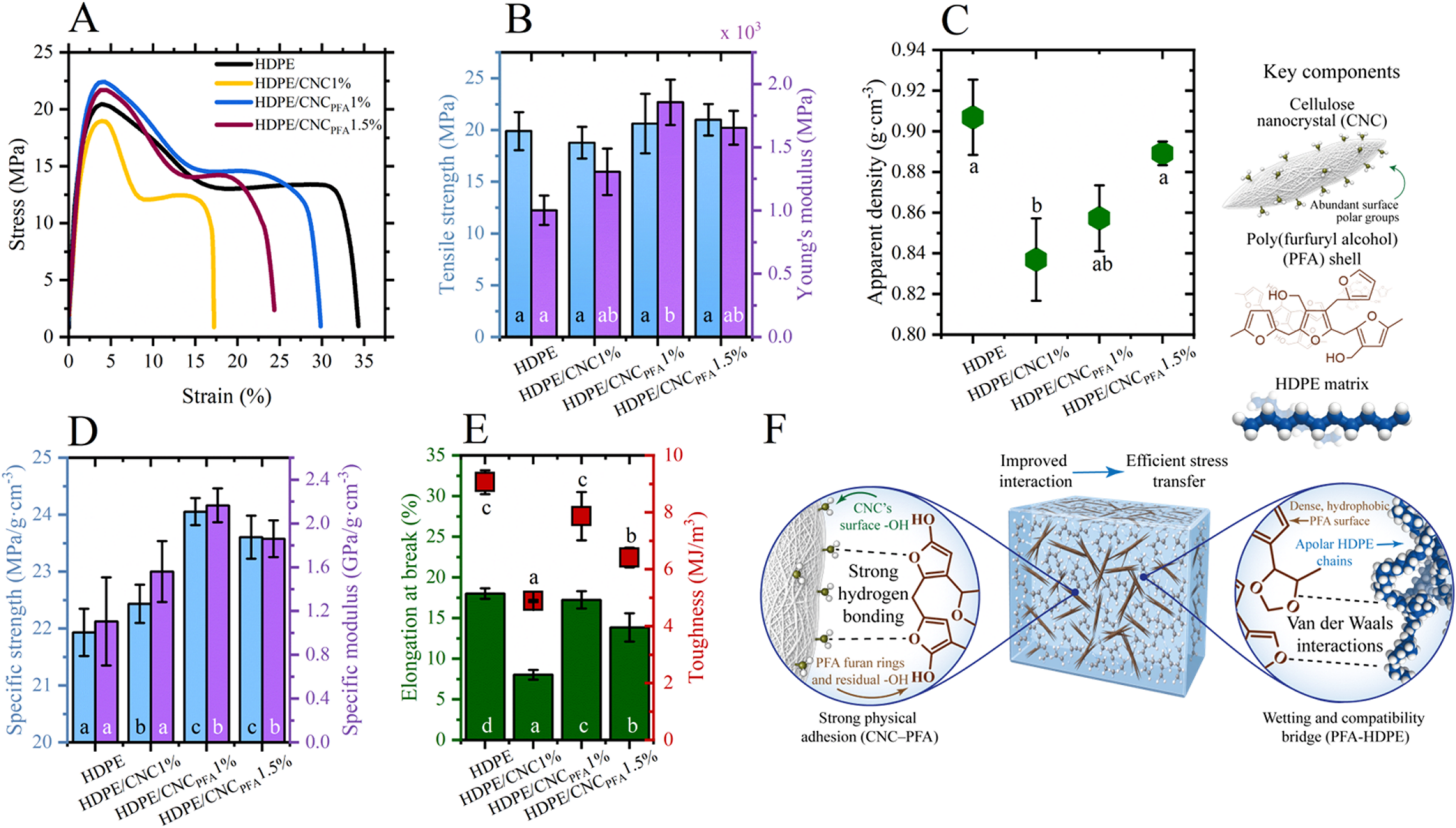
Mechanical properties, apparent density, and compatibilization mechanism of the nanocomposites. (A) Representative tensile stress–strain curves. (B) Tensile strength and Young’s modulus. (C) Apparent density. (D) Specific tensile strength and specific modulus. (E) Elongation at break and toughness. (F) Schematic illustration of the core–shell architecture and interfacial interactions of the nanocomposite. Error bars represent the standard deviation (SD) calculated from independent replicates. Different lowercase letters on the bars indicate statistically significant differences among the sample groups.

The tensile strengths of the nanocomposites are displayed in Figure 6B. According to the ANOVA, the F ratio was 0.99851, while the *p*-value was 0.4296. Since the *p*-value was greater than 0.05, there were no significant differences between the tensile strength means of the four composites at a significance level of 5%. Therefore, statistically significant differences were not identified among the produced samples. This indicates that neither the incorporation of CNCs nor the PFA surface modification significantly altered the tensile strength of the HDPE matrix. While the HDPE/CNCPFA1% group showed a higher numerical average compared to neat HDPE, this variation is reported as a nonsignificant trend rather than a statistical improvement. In a similar study, Pöllänen et al. (2013) used various concentrations of MCC as reinforcement in a HDPE composite compatibilized with PE-*g*-MA. At the 5% MCC concentration, which is closest to that used in the present study, an average increase of 49.5% in tensile strength was detected. The difference in their finding and those of the present manuscript resides on the natural of the reinforcement used: while MCC is able to support appropriate mechanical strength transference from matrix, CNCs do not behave as ideally, thanks to their intrinsic shape. Vahidi et al. (2022), on the other hand, studied the incorporation of CNCs and zinc oxide (ZnO) in an HDPE matrix with PE-*g*-MA, finding a value of 22.1 ± 4.3 for pure HDPE. A progressive decrease was noted with the increase in CNC and ZnO contents: the HDPE-MA-3CNCs-3ZnO composite recorded 20.6 ± 2.6 MPa, while the HDPE-MA-5CNCs-5ZnO exhibited 20 ± 2.8 MPa.

Also in Figure 6B, the Young’s modulus exhibited a general trend of growth up to the HDPE/CNC_PFA_1% composite (1856 MPa), decreasing for the HDPE/CNC_PFA_1.5% composite (1654 MPa). The Young’s modulus, or elasticity, is a measure of a material’s capacity for elastic deformation when subjected to mechanical stress, indicating the resistance of a material to elastic deformation.^58^ In this sense, the PFA impregnated in CNCs demonstrated the ability to increase the rigidity of the nanocomposites (comparing the means of HDPE/CNC1% and HDPE/CNC_PFA_1%). This performance aligns with the nature of PFA itself, a thermosetting substance characterized by the presence of cross-links between its macromolecules, which naturally imparts greater rigidity to the material.^59^ From an engineering perspective, more resilient materials (i.e., those that undergo more elastic deformation before returning to their original state without failing) are often favored for applications in structural components within the automotive industry, civil construction, and sporting equipment.^60^

To further elucidate the effectiveness of the surface modification, a comparative analysis of the reinforcement efficiency was conducted by calculating the normalized Young’s modulus *E*_c_/*E_m_* for the composites at identical filler loadings. For the unmodified HDPE/CNC blends at a 1 wt % loading, the normalized modulus was restricted to 1.30, reflecting the poor stress transfer caused by thermodynamic incompatibility and interfacial microvoids. In stark contrast, the incorporation of the CNC_PFA_ nanostructures at the exact same mass fraction resulted in a significantly higher normalized modulus of 1.85. This substantial enhancement in the reinforcement efficiency factor quantitatively demonstrates the success of the *in situ* modification.

The macroscopic mechanical reinforcement, evidenced by the 42% increase in Young’s modulus for the modified composites, is intrinsically linked to the enhanced interfacial dynamics and stress transfer mechanisms provided by the PFA core–shell encapsulation. In the unmodified HDPE/CNC systems, the severe polarity mismatch prevents adequate matrix wetting, leading to the formation of large interfacial voids (corroborated by SEM) where the rigid agglomerates act as stress concentrators, ultimately compromising the elastic response. By physically enveloping the nanocrystals in a hydrophobic PFA network, the thermodynamic compatibility with the nonpolar polyolefin matrix is drastically improved. Although the single-screw extrusion process inherently leaves residual macro-agglomerates, the robust interfacial adhesion established by the PFA shell effectively eliminates these critical nonadhered boundaries. This continuous and bonded interface facilitates a highly efficient stress transfer mechanism, allowing the applied mechanical load to be effectively transferred from the ductile HDPE matrix into the highly stiff, crystalline cellulose core, thereby directly driving the substantial enhancement in the overall stiffness of the nanocomposite. To contextualize the significance and novelty of these findings, it is imperative to compare the mechanical performance of the developed nanocomposites with previous literature reports on cellulose-reinforced polyolefins. Traditionally, achieving substantial stiffness and toughness improvements in CNC-reinforced HDPE composites requires the addition of fossil-based synthetic coupling agents, such as maleated polyethylene. For instance, while earlier studies like Vahidi et al.^57^ reported progressive decreases in tensile properties when incorporating unmodified CNCs even with a coupling agent, and others achieved stiffness gains only through high filler loadings of microcrystalline cellulose alongside synthetic compatibilizers,^56^ the present study demonstrates a remarkable 85% increase in Young’s modulus over neat HDPE at an exceptionally low filler loading of just 1%. The novelty of the current results lies in achieving these highly competitive mechanical reinforcement levels entirely through a biobased, noncovalent physical encapsulation strategy.

Figure 6C displays the apparent density values of the nanocomposites. For this property, the F ratio was 413.964. Since the *p*-value was 0.048, which is less than 0.05, there is a statistically significant difference between the means of the four variables at a significance level of 5%. While HDPE exhibited an average apparent density of 906.92 kg·m^–3^, all other composites had a lower average density. The density of CNCs is usually close to 1600 kg·m^–3^,^61^ although this value can vary depending on the size of the crystals, which is highly variable depending on the method used for CNC extraction.

The HDPE/CNC1% composite had the lowest average density (836.90 kg·m^–3^), while the furfurylation treatment of CNCs caused increases in this physical characteristic. The experimental fluctuations in the apparent density of the nanocomposites are strictly governed by the quality of the filler–matrix interface and the resulting internal porosity, rather than variations in the molecular weight or intrinsic density of the modifier phase. While the theoretical density of a fully consolidated composite follows a weighted rule of mixtures of its individual components, experimental deviations typically arise from interfacial processing defects. For the unmodified HDPE/CNC1% composite, the sharp decline in apparent density is attributed to the formation of extensive microvoids and interfacial gaps caused by the thermodynamic incompatibility and poor wetting between the hydrophilic CNCs and the nonpolar HDPE matrix. Conversely, the physical encapsulation of the nanocrystals within the hydrophobic PFA shell eliminates these internal air pockets by promoting a continuous and tightly bonded interface, which allows the matrix to fully infiltrate the filler clusters during melt processing and justifies the recovery of the composite’s bulk density.

Figure 6D presents the normalized tensile strength of the nanocomposites, a ratio between tensile strength and the apparent density of the produced materials. In materials science, specific properties are important values that serve as merit indices for material selection, enabling ranking.^62^ Although no significant differences in tensile strength among the composites were detected, the specific tensile strength reveals that there are gains in this attribute when considering variations in the apparent density of the materials. In the graph, the highest specific strength was found for the HDPE/CNC_PFA_1% composite (2.41 MPa/g·cm^–3^), which decreased with the increase in CNC_PFA_ concentration (2.36 MPa/g·cm^–3^). This phenomenon reinforces the idea that, in terms of mechanical properties, the limiting content of CNCs for maximizing tensile strength is 1% of CNC_PFA_. Similarly, the specific modulus also demonstrated a highly favorable increase. Since the elastic modulus increased significantly without penalizing the mass of the composite, the elevated specific modulus of the HDPE/CNC_PFA_1% consolidates the effectiveness of the treatment in creating stiff and lightweight materials, which are imperative attributes for replacing structural components in the automotive and packaging industries.

To elucidate the macroscopic origins of the structural improvements, the core–shell mechanism and the interfacial dynamics are outlined in Figure 6F. The previously discussed substantial enhancements, detailed in the schematic illustration, occurs because the core–shell architecture resolves the polarity conflict in the system. Around the cellulose particle, a very strong adhesion via hydrogen bonding is developed (CNC–PFA inner interface), supported by the furan rings and residual hydroxyls/polar groups from PFA polymerization. Externally, the dense organic PFA surface exhibits excellent wetting and thermodynamic compatibility with the apolar HDPE chains through van der Waals interactions.^63^ By filling the gaps between the matrix and the reinforcement and forming this continuous boundary, the mechanical load is efficiently transferred from the ductile polyethylene matrix directly to the highly stiff cellulose crystals, which translates into the substantial increases in modulus, toughness, and specific properties observed graphically.

The efficacy of the surface modification and the quality of the interfacial interaction between the dispersed phase and the matrix were investigated through the analysis of cryo-fractured surfaces using SEM, as illustrated in Figure 7.

**Figure 7:**
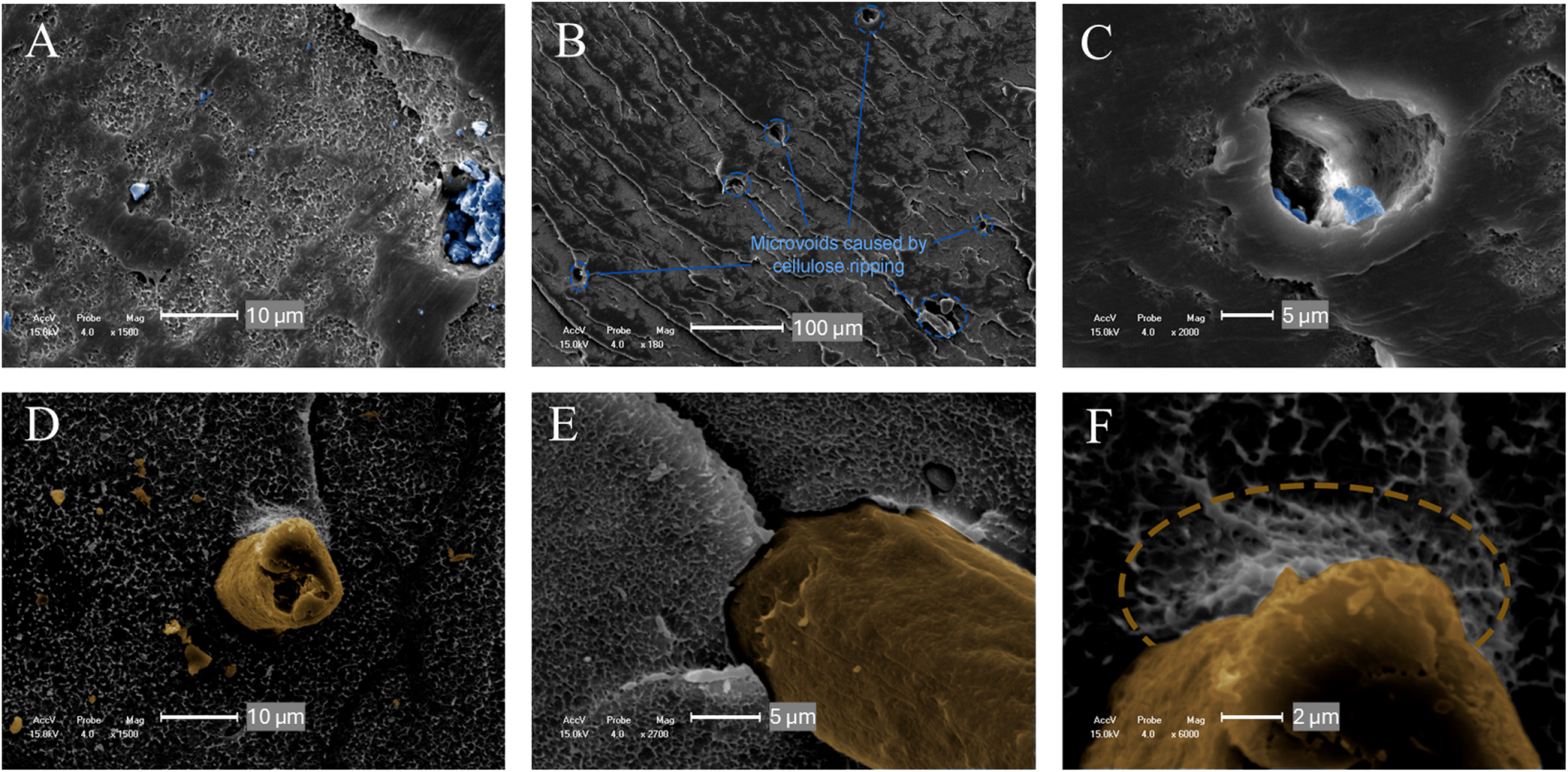
SEM micrographs of cryo-fractured surfaces of the nanocomposites. (A–C) HDPE reinforced with CNCs (HDPE/CNC1%). The arrows and circles in (B) highlight microvoids and cavities. (D–F) HDPE reinforced with PFA-modified CNCs (HDPE/CNC_PFA_1%).

The micrographs referring to the composite reinforced with unmodified nanocrystals (HDPE/CNC1%), presented in Figure 7A–C, evidence a weak interfacial adhesion characteristic of incompatible systems. It is possible to observe the generalized presence of microvoids and deep cavities, highlighted by the arrows and circles in Figure 7B. These structures are indicative of the fiber pull-out phenomenon, which occurs when the interfacial shear strength is insufficient to transfer the load from the matrix to the reinforcement, resulting in the detachment of the particle during fracture. The thermodynamic incompatibility between the nonpolar HDPE matrix and the hydrophilic surface of the CNCs favors not only detachment but also the formation of agglomerates and air entrapment. The presence of these cavities and the associated porosity (Figure 7C) corroborate the reduction in apparent density observed for this formulation, as the lack of effective wettability prevents the complete filling of the interface by the molten polymer.^64^

In contrast, the micrographs of the composite containing modified nanocrystals (HDPE/CNC_PFA_1%), displayed in Figures 7D–F, demonstrate the success of PFA incorporation. The modification via *in situ* polymerization formed a PFA shell around the nanocrystals, as evidenced by the interfacial region highlighted in Figure 7F. This layer acts by shielding the superficial hydroxyl groups of the cellulose, conferring a hydrophobic character to the reinforcement and promoting superior chemical compatibility with the polyethylene matrix. Visually, Figure 7D[Fig fig7],E show that the modified particles are well-anchored and wetted by the polymeric matrix, with a notable absence of the pull-out voids observed in the untreated samples. The structural continuity at the interface suggests that the PFA layer formed an effective interpenetrating physical network, eliminating the formation of bubbles due to poor interaction and allowing for more efficient stress transfer. This mechanism explains the significant increase in Young’s modulus and specific strength reported for this material, consistent with the reinforcement effects observed in other PFA-modified cellulose systems.^65^ Although a direct quantitative calculation of the bulk void fraction from the 2D SEM micrographs is severely limited by the inherent macro- and microheterogeneity of the cryo-fractured surfaces, the morphological evidence clearly substantiates the presence of extensive internal porosity. While a direct quantitative analysis of filler dispersion and interparticle spacing within the consolidated HDPE matrix is restricted by the inherent three-dimensional heterogeneity of the composite fracture planes, the high-resolution particle distributions—combined with the qualitative morphological evidence of microagglomeration from cross-sectional SEM—provide a consistent understanding of the material’s internal architecture and its direct correlation with the macroscopic mechanical performance.

The successful compatibilization of the HDPE/CNC_PFA_ nanocomposites is governed by a hierarchical, noncovalent interfacial mechanism operating at two distinct boundaries. At the inner interface, the interaction between the crystalline core and the polymer shell is dominated by strong secondary supramolecular forces—specifically, extensive hydrogen bonding between the intrinsic surface hydroxyl groups of the cellulose and the oxygen atoms of the furan rings within the highly cross-linked PFA network—combined with the mechanical interlocking of the physical encapsulation. At the outer boundary, the PFA shell serves as a critical thermodynamic bridge. By physically masking the highly polar, hydrophilic cellulose surface, the significantly more hydrophobic PFA coating dramatically reduces the polarity gap and interfacial tension with the nonpolar HDPE matrix. Consequently, the compatibility between the modified filler and the polyolefin is driven by enhanced physical wetting, van der Waals forces, and macromolecular chain entanglement during melt extrusion. This physically compatibilized architecture effectively minimizes the formation of nonadhered interfacial microvoids, facilitating the efficient macroscopic stress transfer confirmed by the mechanical characterization.

Figure 8 reveals the thermogravimetric behavior of the produced nanocomposites using cellulosic raw materials with the thermogravimetric profile discussed earlier as reinforcement. For these materials, the addition of CNCs caused little alteration, due to the low filler contents used. As a result, the thermal degradation observed for the composites primarily reflects the degradation profile of the HDPE itself.

**Figure 8:**
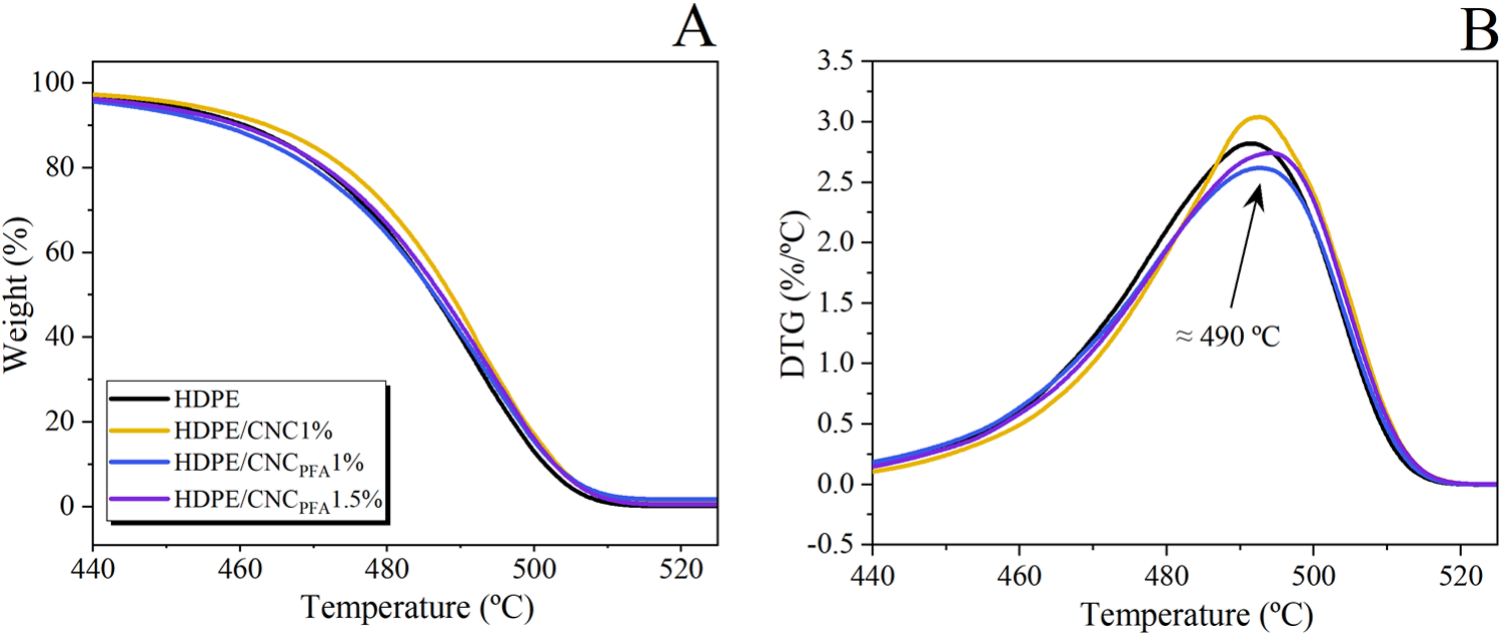
TGA (A) and DTG (B) curves of the nanocomposites.

Up to 440 °C, little mass loss is recorded. Between 440 and 510 °C, the main thermogravimetric event of the nanocomposites occurs. The almost complete mass volatilization at this stage is due to the generation of gaseous products in the form of hydrogen, alkenes (C_2_–C_4_), alkanes (C_1_–C_5_), 1,3-butadiene, and CO.^66^ The formation of these products occurs through the progressive homolytic chain cleavage, leading to the generation of free radicals that react and are stabilized through inter- or intramolecular chain transfers. Moreover, polymeric radicals are formed and stabilized through β cleavages, constituting unsaturated molecules.^67^

## 4 Conclusions

The in situ polymerization of FA onto CNCs proved to be a feasible and sustainable strategy to enhance interfacial adhesion in HDPE-based nanocomposites. Morphological and spectroscopic analyses, particularly TEM and NMR, confirmed the formation of a hydrophobic PFA shell encapsulating the CNCs. This modification was driven primarily by the formation of a physical interpenetrating network rather than covalent etherification, effectively shielding the hydrophilic cellulose surface. Despite the fact that specific dynamic assessments such as rheology or DMA were not performed, the morphological evidence indicates that the nanoscale dispersion of the reinforcement was not fully ideal. The presence of residual macro-agglomerates is a direct consequence of the processing methodology, which utilized a single-screw extruder without prior high-shear compounding equipment, thereby limiting the dispersive mixing capabilities. Remarkably, despite this suboptimal dispersion, the CNC_PFA_ composites still exhibited significant improvements in their macroscopic mechanical properties compared to the unmodified blends. The composite reinforced with 1% PFA-modified CNCs (HDPE/CNC_PFA_1%) exhibited the highest specific strength among all samples (24.1 MPa/g·cm^–3^). Notably, the inclusion of PFA significantly enhanced the material’s stiffness: the Young’s modulus reached 1856 MPa, representing an increase of approximately 42% compared to the unmodified CNC composite and nearly 85% compared to neat HDPE. Furthermore, at a 1.5% concentration, the modified nanocrystals acted as effective nucleating agents, promoting an increase in the *X*_c_ of the HDPE matrix. This finding suggests that the robust interfacial adhesion and physical compatibilization provided by the hydrophobic PFA core–shell encapsulation effectively compensate for dispersive processing limitations, enabling efficient stress transfer even under nonideal industrial mixing conditions. This study successfully establishes a proof-of-concept for the use of PFA as a highly effective physical compatibilizer and thermal shield for nanocellulose in polyolefin matrices. While the physical core–shell encapsulation decisively improved mechanical and thermal properties of the nanocomposites at a 1.5 wt % loading compared to unmodified blends, future systematic studies evaluating a broader range of filler concentrations will be essential to statistically map the reinforcement trends and determine the optimal loading threshold for specific structural applications of these advanced biocomposites.

## Supplementary Material



## Data Availability

The research data is illustrated in the tables and figures provided. Additional information can be acquired by contacting the corresponding author, subject to a reasonable request.

## Notes

FEG-SEM micrographs of the isolated unmodified CNCs and PFA-modified cellulose nanocrystals (PDF)
