# Supplementary material for: Poly(furfuryl alcohol) as a Surface Modifier for Cellulose Nanocrystals Reinforced HDPE Nanocomposites
Source: Biomacromolecules. 2026 Jul 7;27(8):5164–78. doi: 10.1021/acs.biomac.6c00329 (PMC13463545; doi:10.1021/acs.biomac.6c00329)
Supplement: Supplementary file 1 [file bm6c00329_si_001.pdf]

Poly(furfuryl alcohol) as a surface modifier for cellulose nanocrystals reinforced HDPE nanocomposites

Marlon Bender Bueno Rodrigues[a*], Mariane Weirich Bosenbecker[a], Amanda Dantas de Oliveira[a], Silvana I. Wolke[b], Willian Cézar Nadaleti[c], Neftali Lenin Villarreal Carreno[a], Rafael de Avila Delucis[a], André Luiz Missio[a*]

[a]Graduate Program in Materials Science and Engineering (PPGCEM), Federal University of Pelotas (UFPel), Pelotas, 96010-610, Brazil;
[b]Chemistry Institute, Federal University of Rio Grande do Sul (UFRGS), Porto Alegre, 91501-970, Brazil;
[c]Graduate Program in Environmental Sciences (PPGCAmb), Federal University of Pelotas (UFPel), Pelotas, 96010-610, Brazil.

Corresponding authors (*): marlon.bender@ufpel.edu.br, andre.missio@ufpel.edu.br.

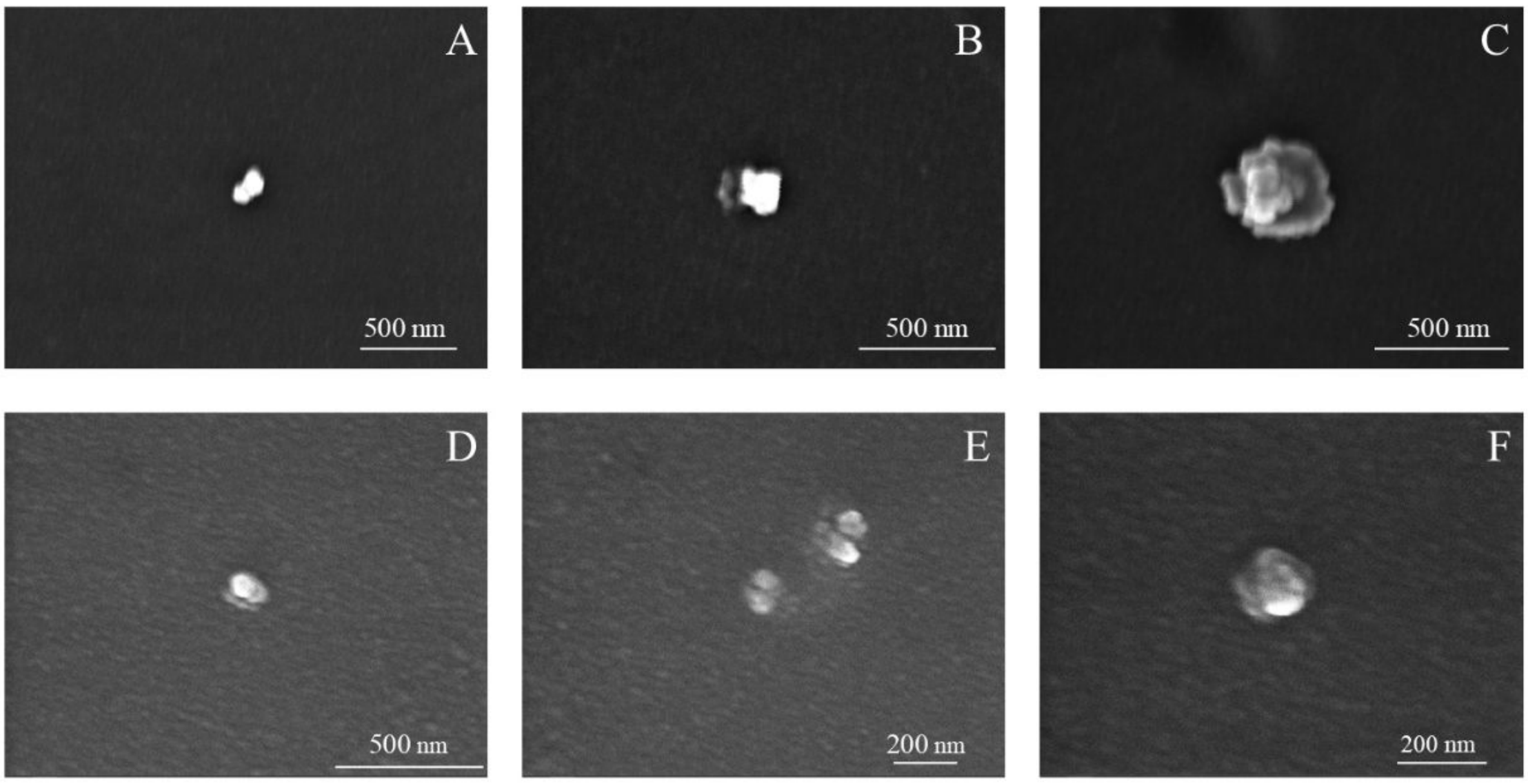

Figure S1. Field emission gun scanning electron microscopy (FEG-SEM) micrographs of the isolated nanoparticles: (A–C) unmodified CNCs and (D–F) CNC_{PFA} at various magnifications.
